# Comparison of the intensity of biofilm formation by *Listeria monocytogenes* using classical culture-based method and digital droplet PCR

**DOI:** 10.1186/s13568-020-01007-5

**Published:** 2020-04-17

**Authors:** Katarzyna Grudlewska-Buda, Krzysztof Skowron, Eugenia Gospodarek-Komkowska

**Affiliations:** Department of Microbiology, Nicolaus Copernicus University in Toruń, Collegium Medicum of L. Rydygier in Bydgoszcz, Bydgoszcz, Poland

**Keywords:** *Listeria monocytogenes*, Droplet digital PCR, Quantification, *hly*A, Biofilm, Abiotic surfaces

## Abstract

*Listeria monocytogenes* is a Gram-positive bacterium, commonly found in food, water or sewage. This microorganism is capable of forming biofilm on different surfaces such as steel, glass, polypropylene etc. Recently an increase in cases of listeriosis has been noted, making *L. monocytogenes* the important health threat. Therefore, there is a need for rapid and sensitive detection of this pathogen. This study aimed to compare the number of *L. monocytogenes* cells recovered from the biofilm (prepared on steel and polypropylene) using the detection and amplification of the *hlyA* gene (droplet digital PCR, ddPCR) and the classical culture method. The research material consisted of 96 *L. monocytogenes* strains. A total of 58 isolates were obtained from clinical samples and 38 isolates derived from the municipal sewage treatment plant. Additionally, the reference strain ATCC^®^19111™ (WDCM00020) was used. The Pearson correlation coefficient for the results obtained by the classical culture-based method and ddPCR was 0.864 and 0.725, for biofilms produced on AISI 304 stainless steel surface and the polypropylene surface, respectively. Correlations were statistically significant (p ≤ 0.001), indicating that the ddPCR technique is an effective tool for the assessment of bacteria number in the biofilm.

## Introduction

*Listeria monocytogenes* is a Gram-positive, facultative anaerobic, non sporulating bacterium, widespread in the environment i.e. soil, water, sewage, faeces of humans and animals (Camejo et al. 2011; Wałecka et al. 2009). *L. monocytogenes* is an etiological factor of human listeriosis. People particularly vulnerable to infection are the elderly, pregnant women and newborns (Schuppler and Loessner 2010). In 2017, the European Food Safety Authority (EFSA) reported 2480 confirmed cases of invasive listeriosis in 28 European Union countries, of which 227 were fatal (EFSA and ECDC 2018). The occurrence of *L. monocytogenes* in wastewater was reported in the 1980s (Al-Ghazali and Al-Azawi 1986). Al-Ghazali and Al-Azawi (1986) demonstrated that *L. monocytogenes* can survive in the sludge for 13 months. *L. monocytogenes* may also be present in the influent wastewater used for the plant treatment (i.e. raw) and wastewater drying process does not lead to its eradication (Al-Ghazali and Al-Azawi 1988). Since contamination of raw sewage with these bacteria can be high the use of sewage sludge as an agricultural fertilizer can be a potential source of soil pollution.(Al-Ghazali and Al-Azawi 1986).

Classic microbiological methods are widely used all over the world, but since they are time-consuming (24 to 48 h), their use in the control of environmental pollution and food is limited (Bian et al. 2015). In addition, early and rapid detection of pathogenic bacteria in the material collected from patients is crucial for the effective treatment of human infections and prevention of epidemic spread. Increasingly, methods based on the analysis of the nucleic acid such as: PCR, real time-PCR (qPCR), DNA microarrays, biosensors (electrochemistry manual, optical), as well as immunological methods are utilized (Mortari and Lorenzelli 2014; Law et al. 2015). The qPCR method is a sensitive and specific technology used to quantify nucleic acids in a sample (Bian et al. 2015). This method requires highly purified DNA template, and a representative sample size (Rossmanith and Wagner 2011). In this technique the DNA amount is proportional to the fluorescence, which is monitored at each cycle of PCR. The point at which the amount of DNA increases and the fluorescence exceeds the background level is called the threshold cycle (CT) or crossing point. By using multiple dilutions of a known amount of standard DNA, a standard curve of concentration in log scale against CT can be generated. The amount of DNA or cDNA in an unknown sample can then be calculated from its CT value. The level of sample contamination affects the effectiveness of qPCR. Samples with a low DNA concentration and high impurity cannot be diluted properly. Additionally, any chemical and/or protein impurities change the Taq polymerase activity and primers binding, which impact the CT values (Taylor et al. 2017). The qPCR technique has been used to determine the level of expression of genes involved in biofilm formation of *L. monocytogenes* (*agr* and *prfA*) (Gandra et al. 2019). Third-generation PCR, known as a droplet digital PCR (ddPCR), allows fast and quantitative detection (Cremonesi et al. 2016). Droplet digital PCR, based on the amplification of a single target DNA molecule in a plurality of separate droplets, enables an exact quantification of DNA copy numbers (Witte et al. 2016b). The principle of ddPCR method is founded on DNA analysis according to the Poisson distribution. The reaction is divided into many small reactions (20,000 drops for the Bio-Rad system) that either contain DNA or not (Hindson et al. 2011). In addition to high precision and sensitivity, ddPCR ensures high reproducibility of the within-laboratory scale (Quan et al. 2018). This method has been used, among others for: routine analysis of genetically modified organisms in food and animal feed (Gerdes et al. 2016); detection and quantification of pathogens such as *Salmonella* spp., *Campylobacter jejuni* and *L. monocytogenes* in the environmental water (Rothrock et al. 2013) and monitoring of the dynamics of the microbial population in soils (Kim et al. 2014). Advantages of ddPCR, compared to qPCR, are the absolute quantification of a target nucleic acid (without the use of calibration curves) (Hindson et al. 2011; Pinheiro et al. 2012), high sensitivity and accuracy for nucleic acids with the low copy number (Sanders et al. 2011; Whale et al. 2012). In turn, the automation of droplet generation in ddPCR method reduces the risk of laboratory errors (Witte et al. 2016a). However, there is still a risk of cross-contamination during droplets transfer. For this reason, fully automatic closed systems have been developed. New capillary-based integrated ddPCR system limits cross-contamination. A HPLC T-junction is used to generate droplets and a long HPLC capillary connects the generator with both a capillary-based thermocycler and a capillary-based cytometer (Chen et al. 2018).

*Listeria monocytogenes* is capable of forming a biofilm on many surfaces, both hydrophilic and hydrophobic, which allows the persistence of the pathogen in the environment, food processing wastewater or sewage (Barbosa et al. 2013). Additionally, in the biofilm structure the bacterium shows higher antimicrobial resistance compared to the planktonic forms (Pan et al. 2006). Currently, several methods are known for the assessment of bacterial adhesion and ability to form biofilm. Among the classical methods, culture methods, microscopic techniques (Chae and Schraft 2000) and spectrophotometric measurement of the biofilm stained with colored substances (crystal violet) (Pan et al. 2006) are used. Also metabolic tests allowing quantification of bacterial viability in the biofilm structure are applied (Gamble and Muriana 2007). The use of ddPCR (Klančnik et al. 2015) is a new approach to assess the cell adhesion in biofilm structure. Ricchi et al. (2017), comparing the application of PCRs (qPCR and ddPCR) with the culture methods for the quantification of *L. monocytogenes* number, have found that PCR may be a valid alternative.

The aim of the study was to assess the number of *L. monocytogenes* cells recovered from the biofilms on steel and polypropylene, based on the detection and amplification of a single copy of *hlyA* gene, using the ddPCR technique. The effectiveness of this method was compared with the classical culture-based method.

## Materials and methods

### Material

The study covered 96 strains of *L. monocytogenes,* of which 58 were isolated from clinical samples and 38 strains were selected from the environment of the municipal sewage treatment plant in Northern Poland. The reference strain ATCC^®^19111™ (WDCM00020) was also included in the study. As this strain was described as the reference strain in the food testing applications, we used it in our study for the evaluation of biofilm formation on surfaces frequently used in the food industry. All strains used in the study are from the collection of the Department of Microbiology, Ludwik Rydygier Collegium Medicum in Bydgoszcz, Nicolaus Copernicus University in Toruń. The origin of the strains is presented in Table 1. Strains derived from the clinical material were isolated in 2005–2017, and environmental strains were obtained in 2013–2014 from the municipal sewage treatment plant. Examined isolates were stored at − 80 °C in BHI broth (Brain Heart Infusion, bioMérieux) with the addition of 15% glycerol (Avantor).

**Table 1:** *Listeria monocytogenes* strains origin

	Origin (n = 96)	Strains number	Number of strains (%)
Clinical sample	Blood	1K, 2K, 3K, 5K, 6K, 7K, 9K, 10K, 19K, 21K, 24K, 28K, 31K, 33K, 35K, 36K, 39K, 40K, 41K, 42K, 43K, 44K, 45K, 46K, 47K, 48K, 51K, 53K, 56K, 57K, 58K	30 (31.2)
	CSF	4K, 11K, 12K, 17K, 20K, 22K, 25K, 26K, 27K, 29K, 34K, 38K, 55K	13 (13.5)
	Vagina	13K, 14K, 15K, 16K, 30K, 32K	6 (6.3)
	Cervix	8K	1 (1.0)
	Descending colon tumor	50K	1 (1.0)
	Heart valve	52K	1 (1.0)
	Blood from catheter	49K	1 (1.0)
	Ear swab	23K	1 (1.0)
	Pharyngeal swab	54K	1 (1.0)
	Peritoneal fluid	18K	1 (1.0)
	Dialysis fluid	37K	1 (1.0)
Sample from sewage treatment plants	Raw sludge	1OS, 2OS, 3OS, 4OS, 5OS, 6OS, 7OS, 8OS, 9OS, 10OS, 11OS, 12OS, 13OS	13 (13.5)
	Sewage (inflow)	1ŚD, 2ŚD, 3ŚD, 4ŚD, 5ŚD, 6ŚD, 7ŚD, 8ŚD, 9ŚD, 10ŚD, 11ŚD, 12ŚD, 15ŚD, 16ŚD, 17ŚD, 18ŚD	16 (16.7)
	Air	1POW, 2POW, 3POW	3 (3.1)
	Stabilized sludge	1OSTAB, 2OSTAB, 3OSTAB, 4OSTAB, 5OSTAB, 6OSTAB	6 (6.3)

*CSF* cerebrospinal fluid

### Genetic similarity

The genetic similarity of selected *L. monocytogenes* strains was determined with the Pulsed-Field Gene Electrophoresis (PFGE). The procedure for genotyping was performed in accordance with the Standard Operating Procedure for PulseNet PFGE of *Listeria monocytogenes* (PNL04, last update April 2014) (PulseNet 2013).

### Isolation of genomic DNA

Isolation of genomic DNA was performed using the Genomic Mini AX Bacteria Spin Kit (A&A Biotechnology), according to the manufacturer’s procedure.

### Classical culture-based method

Assessment of the biofilm formation by the examined *L. monocytogenes* strains was performed on sterile parts of stainless steel ASI 304 (BTH Import Stal) and polypropylene (Quadrant EPP Poland Sp.) with dimensions of 1 × 2 cm and a thickness of 1 mm. The intensity of biofilm formation was determined as previously described (Skowron et al. 2019).

The tested strains were plated onto Columbia agar with 5% sheep blood (CAB) (bioMérieux) and incubated for 24 h at 37 °C. The grown colonies were used to prepare 3 ml of the suspension (0.5 of MacFarland scale) in BHI (Becton–Dickinson). Sterile polypropylene or stainless steel fragments were placed in the prepared bacterial suspensions and incubated at 37 °C for 24 h. The negative control constituted sterile parts of polypropylene and stainless steel incubated in sterile BHI medium in the same conditions. Then the fragments were washed with sterile phosphate buffered saline (PBS) pH 7.2 (Avantor) and placed in sterile BHI medium. This operation was repeated after another 24 h of incubation. After this time, the surfaces were washed twice with PBS (pH 7.2) to remove the planktonic cells. Then the samples were sonicated (Ultrasonic DU-4, Nickel-Electro) with the following parameters: operating frequency—30 kHz, power—150 W, temperature 25 °C for 10 min and shaking (500×*g*) for 10 min. A serial tenfold dilutions (10^−6^) in PBS (pH 7.2) of bacterial suspensions were made and plated onto CAB agar (0.1 ml). After 24 h of aerobic incubation at 37 °C the number of colonies was counted. The results were presented as the number of colony forming units per 1 cm^2^ surface (CFU × cm^−2)^ of ASI 304 stainless steel or polypropylene.

### Digital PCR droplet

In the first stage of the study, the biofilm was prepared according to the procedure described above. The sonicated samples were shaken and centrifuged (9700×*g*, 5 min) and then for each sample DNA was isolated. The quality and concentration of DNA was checked using the BioPhotometer D30 spectrophotometer (Eppendorf).

The ddPCR method was performed using the QX200™ Droplet Digital PCR (ddPCR™) system (Bio-Rad). A region of *L. monocytogenes* listeriolysin O gene (*hly*A) was used as a target for PCR amplification. The forward primer (5ʹ-ACTGAAGCAAAGGATGCATCTG-3ʹ) and the reverse primer (5ʹ-TTTTCGATTGGCGTCTTAGGA-3ʹ) were used to amplify a 106-bp segment of the *hly*A gene (Suo et al. 2010). This gene is constitutive and species-specific for *L. monocytogenes*.

A 22 µl of reaction mix contained: 11 µl of QX200™ ddPCR™ EvaGreen Supermix (Bio-Rad), 0.22 µl of each primer (100 µM) (Oligo.pl), 1 µl of genomic DNA and ultra pure water. The reaction mixture was then placed in a drop generator (QX200™ Droplet Generator) (Bio-Rad) to produce, after combining with the oil, about 20,000 drops of equal volume and size (QX200™ Droplet Generation Oil for EvaGreen) (Bio-Rad). The control of the drop generation process was a reaction mixture containing ultra-pure water instead of DNA.

Reaction was performed in a thermocycler C1000 Touch™ Thermal Cycler (Bio-Rad) with the following conditions for the amplification: initial denaturation (95 °C/5 min), 40 cycles of denaturation (95 °C/30 s), annealing (58 °C/30 s) and elongation (72 °C/1 min) and the final elongation (72 °C/10 min). The PCR products were denatured at 98 °C for 10 min and kept at 4 °C until the droplets were read.

In the next step, plates were placed in a drop reader (Bio-Rad) and the results were read determining the cut-off point. The fluorescence amplitude threshold, used for the discrimination of the positive and negative droplets in QuantaSoft software was set between 7000 and 8000. This value was chosen after optimization of the probe concentration and annealing temperature in the ddPCR assay. The concentration values were calculated using QuantaSoft software (in copies μl^−1^) and multiplied by 22 (the initial PCR volume) to obtain the absolute number of copies added to the reaction.

### Statistical analysis

The analysis of the obtained results was carried out using STATISTICA 13.1 PL (StatSoft) program based on the Tukey post hoc test. The differences were considered statistically significant at the probability level of p < 0.05. The correlation between the intensity of biofilm formation, assessed by the classical culture-based method, and ddPCR was made using the interpretation of the Pearson correlation coefficient in relation to the Guillford scale. Correlations were considered statistically significant for the test probability p ≤ 0.05.

## Results

### Genetic similarity of *Listeria monocytogenes* strains

**Fig. 1:**
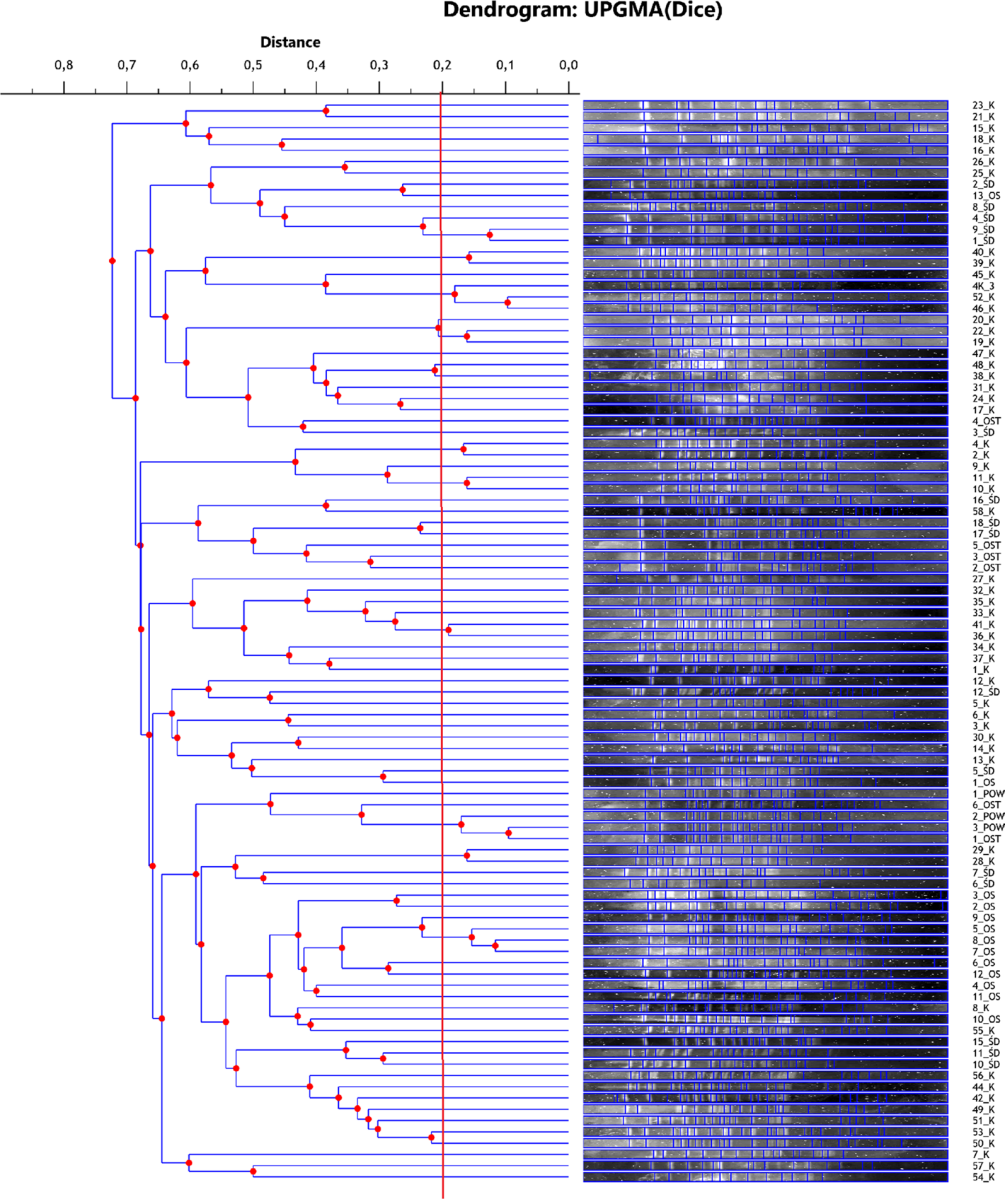
Dendrogram of ApaI-PFGE cluster analysis of 96 *L. monocytogenes* isolates with sample identification data. 1_K-58_K, clinical samples; 1_OS-13_OS; 1_ŚD-18_ŚD;1_OSTAB-6_OSTAB; 1_POW-3_POW, environmental samples

All strains used in experiment were genetically different (Fig. 1). The cut-off value to define the PFGE patterns was set at 80% similarity. For the cut-off at level of 80%, 3 clusters (C3, C8 and C10) containing 3 strains and 7 clusters (C1, C2, C4, C5, C6, C7 and C9) containing 2 strains were found (Table 2). With the exception of the C8 cluster, all clusters included strains isolated from the same material. Cluster C1 included strains originating from the inflowing sewage, cluster C10—strains from the raw sludge, and the other clusters—strains derived from blood. Cluster C8 included 1 strain isolated from the stabilized sludge and 2 strains derived from the air of the wastewater treatment plant (Table 2). Together, 23 strains were classified into similarity clusters. The rest of strains showed no genetic similarity and were not included in any cluster.

**Table 2:** The cluster of genetic similarity determined for tested strains at cut-off level of 80%

PFGE cluster	Isolate number
C1	1_ŚD, 9_ŚD
C2	39_K, 40_K
C3	43_K, 46_K, 52_K
C4	19_K, 22_K
C5	2_K, 4_K
C6	10_K, 11_K
C7	33_K, 41_K
C8	1_OST, 2_POW, 3_POW
C9	28_K, 29_K
C10	5_OS, 7_OS, 8_OS

### Classical culture-based method

**Fig. 2:**
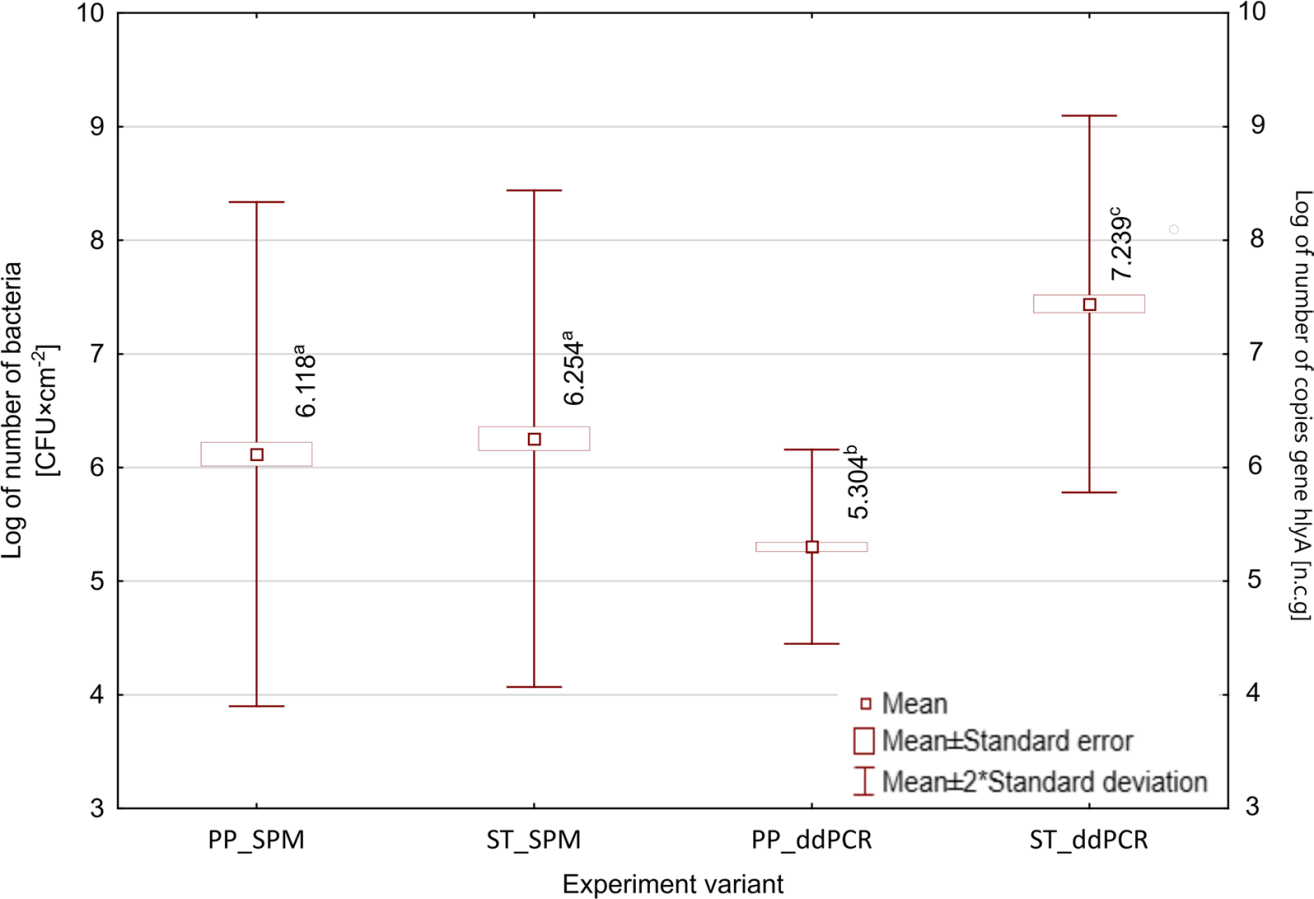
Differences in the intensity of biofilm formation by the tested *L. monocytogenes* strains on the surface of polypropylene and stainless steel. a, b, c,… - values marked with different letters are significantly different (p ≤ 0.05)

The obtained results allowed to observe differences in the intensity of biofilm formation on the surface of polypropylene and ASI 304 stainless steel by the tested strains of *L. monocytogenes*. The calculated differences in the intensity of biofilm formation between two tested surfaces were not statistically significant (p > 0.05) (Fig. 2).

The number of bacteria reisolated from polypropylene ranged from 4.26 log CFU × cm^−2^ to 7.97 log CFU × cm^−2^. The recovery of the cells from the biofilm formed on fragments of polypropylene was 5.64 log CFU × cm^−1^ for the reference strain ATCC^®^19111™ (WDCM00020). The biofilm formation intensity on the polypropylene surface varied between the tested *L. monocytogenes* strains (Table 3). It was found that strains weakly forming biofilm were statistically significantly (p ≤ 0.05) more often among strains isolated from the clinical material. On the other hand, very strong biofilm-formers were statistically significantly (p ≤ 0.05) more frequently isolated from the environment of sewage treatment plants (Table 3).

**Table 3:** Classification of tested *L. monocytogenes* rods depending on the biofilm formation intensity based on the number of bacteria isolated from the polypropylene surface in a quantitative method

Intensity of biofilm formation by *L. monocytogenes*	Number of reisolated *L. monocytogenes* [log CFU × cm^−2^]	Number of strains depending on the origin [n (%)]		p value^a^	Total n = 96
		Clinical strains n = 58	Environmental strains n = 38		
Weak	4.26–5.29	19 (31.0)	6 (15.8)	*0.011*	24 (25.0)
Moderate	5.30–5.91	15 (27.6)	8 (21.1)	0.284	24 (25.0)
Strong	5.92–6.85	13 (22.4)	11 (28.9)	0.293	24 (25.0)
Very strong	6.86–7.97	11 (19.0)	13 (34.2)	*0.015*	24 (25.0)

Italic values indicate significance of p value (p ≤ 0.05)

^a^p value ≤ 0.05

The number of bacteria reisolated from the steel surface ranged from 4.28 log CFU × cm^−2^ to 7.90 log CFU × cm^−2^. For the reference strain ATCC^®^19111™ (WDCM00020) the number of recovered bacteria was 5.98 log CFU × cm^−1^. The tested isolates showed different strength of biofilm formation on the steel surface (Table 4). The strains of weak and moderate biofilm formation ability were statistically significantly (p ≤ 0.001) more often found among the strains isolated from the sewage treatment plant. In contrast, strains strongly and very strongly forming biofilm on the steel surface were statistically significantly (p ≤ 0.001) more frequently detected among the clinical strains (Table 4).

**Table 4:** Classification of tested *L. monocytogenes* rods depending on the biofilm formation intensity based on the number of bacteria isolated from the steel surface in a quantitative method

Intensity of biofilm formation by *L. monocytogenes*	Number of reisolated *L. monocytogenes* [log CFU × cm^−2^]	Number of strains depending on the origin [n (%)]		p value	Total n = 96
		Clinical strains n = 58	Environmental strains n = 38		
Weak	4.28–5.25	6 (10.3)	18 (47.4)	p < 0.001	24 (25.0)
Moderate	5.26–5.87	7 (12.1)	17 (44.7)	p < 0.001	24 (25.0)
Strong	5.88–6.80	21 (36.2)	3 (7.9)	p < 0.001	24 (25.0)
Very strong	6.81–7.90	24 (41.4)	0 (0.0)	p < 0.001	24 (25.0)

### ddPCR

The number of copies of the *hlyA* gene in samples from the polypropylene surface ranged from 4.53 log ncg to 6.40 log ncg. For the reference strain ATCC^®^19111™ (WDCM00020) the number of the *hlyA* gene copies was 5.28 log ngc (polypropylene). The correlation between strains origin and biofilm intensity was noted. The strains of weak biofilm forming ability were statistically significantly (p ≤ 0.001) more often isolated from the clinical material. On the other hand, strains that strongly formed biofilm on the examined surface were statistically significantly (p ≤ 0.001) more frequently isolated from the environment of sewage treatment plants (Table 5).

**Table 5:** Classification of tested *L. monocytogenes* rods depending on the biofilm formation ability on the polypropylene surface based on the number of copies of the *hlyA* gene assessed by droplet digital PCR

Intensity of biofilm formation by *L. monocytogenes*	Number of copies gene *hlyA* [log n.g.c^a^]	Number of strains depending on the origin [n (%)]		p value	Total n = 96
		Clinical strains n = 58	Environmental strains n = 38		
Weak	4.53–4.92	19 (32.8)	5 (13.2)	p < 0.001	24 (25.0)
Moderate	4.93–5.29	16 (27.6)	8 (21.1)	0.284	24 (25.0)
Strong	5.30-5.59	14 (24.1)	10 (26.3)	0.720	24 (25.0)
Very strong	5.60–6.40	9 (15.5)	15 (39.5)	p < 0.001	24 (25.0)

^a^Number of copies gene *hlyA*

The number of copies of the *hlyA* gene in samples from the stainless steel surface ranged from 4.55 log ncg to 8.46 log ncg. For the reference strain ATCC^®^19111™ (WDCM00020) the number of copies of the *hlyA* gene was 6.79 log ngc (steel). The study showed a diversified biofilm formation ability of the tested *L. monocytogenes* bacilli. It was observed that the strains weakly and moderately forming biofilm occurred statistically significantly (p ≤ 0.001) more often among strains isolated from the sewage treatment plant environment. In turn, the strains strongly and very strongly forming biofilm on the examined surface were statistically significantly (p ≤ 0.001) more often found in the clinical material (Table 6).

**Table 6:** Classification of tested *L. monocytogenes* rods depending on the strength of biofilm formation on the steel surface based on the number of copies of the *hlyA* gene assessed by droplet digital PCR

Intensity of biofilm formation by *L. monocytogenes*	Number of copies gene *hlyA* [log n.g.c^a^]	Number of strains depending on the origin [n (%)]		p value	Total n = 96
		Clinical strains n = 58	Environmental strains n = 38		
Weak	4.55–6.93	6 (10.3)	17 (44.7)	p < 0.001	24 (25.0)
Moderate	6.94–7.42	7 (12.1)	20 (52.6)	p < 0.001	24 (25.0)
Strong	7.43–8.05	21 (36.2)	1 (2.6)	p < 0.001	24 (25.0)
Very strong	8.06–8.46	24 (41.4)	0 (0.0)	p < 0.001	24 (25.0)

^a^Number of copies gene *hlyA*

### Comparison of the intensity of biofilm formation by *L. monocytogenes* strains assessed by classical culture-based and ddPCR methods

**Fig. 3:**
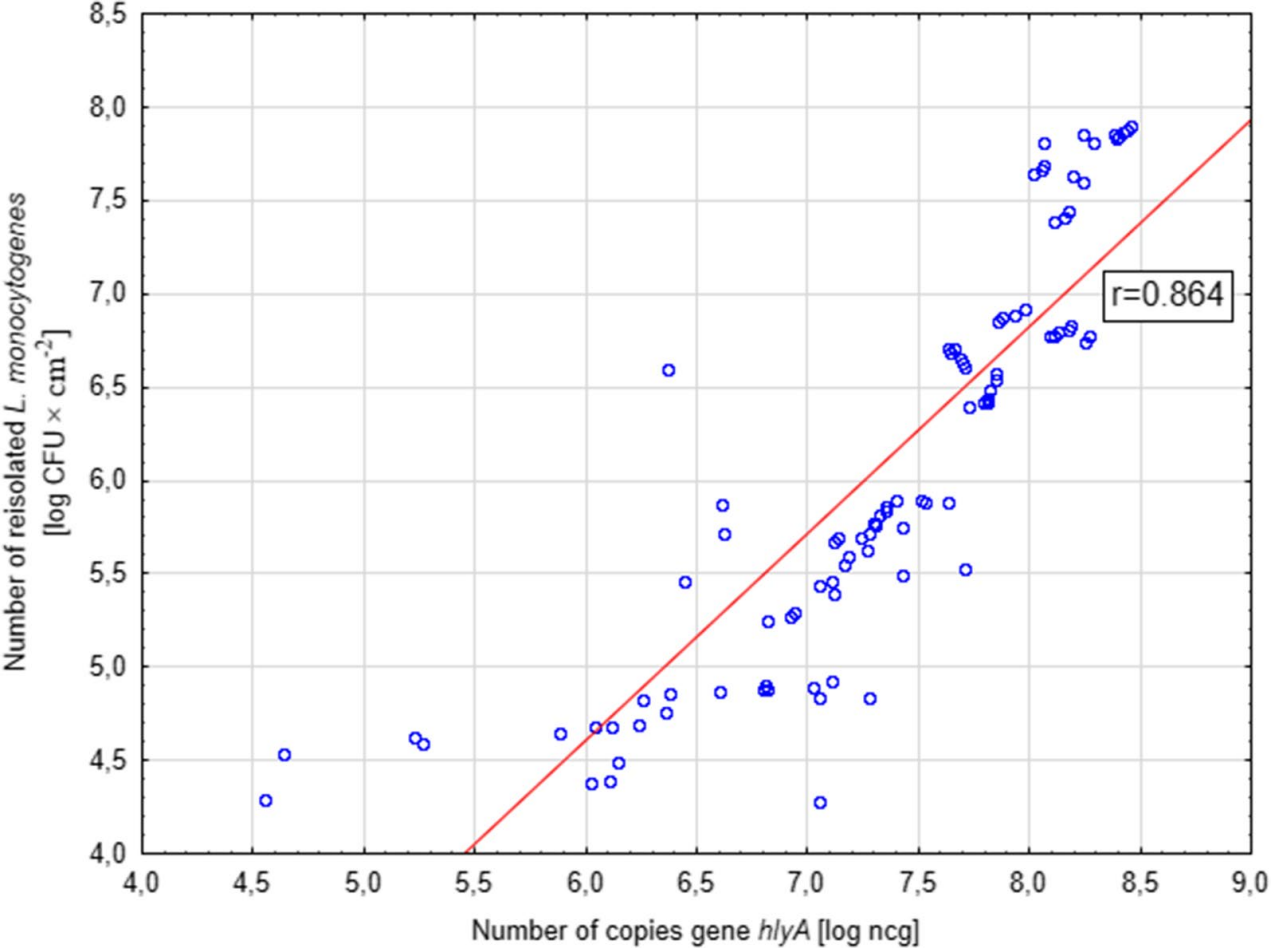
Correlation between the number of bacteria reisolated from the polypropylene surface and the number of copies of the *hlyA* gene in the sample for the tested strains of *L. monocytogenes*

**Fig. 4:**
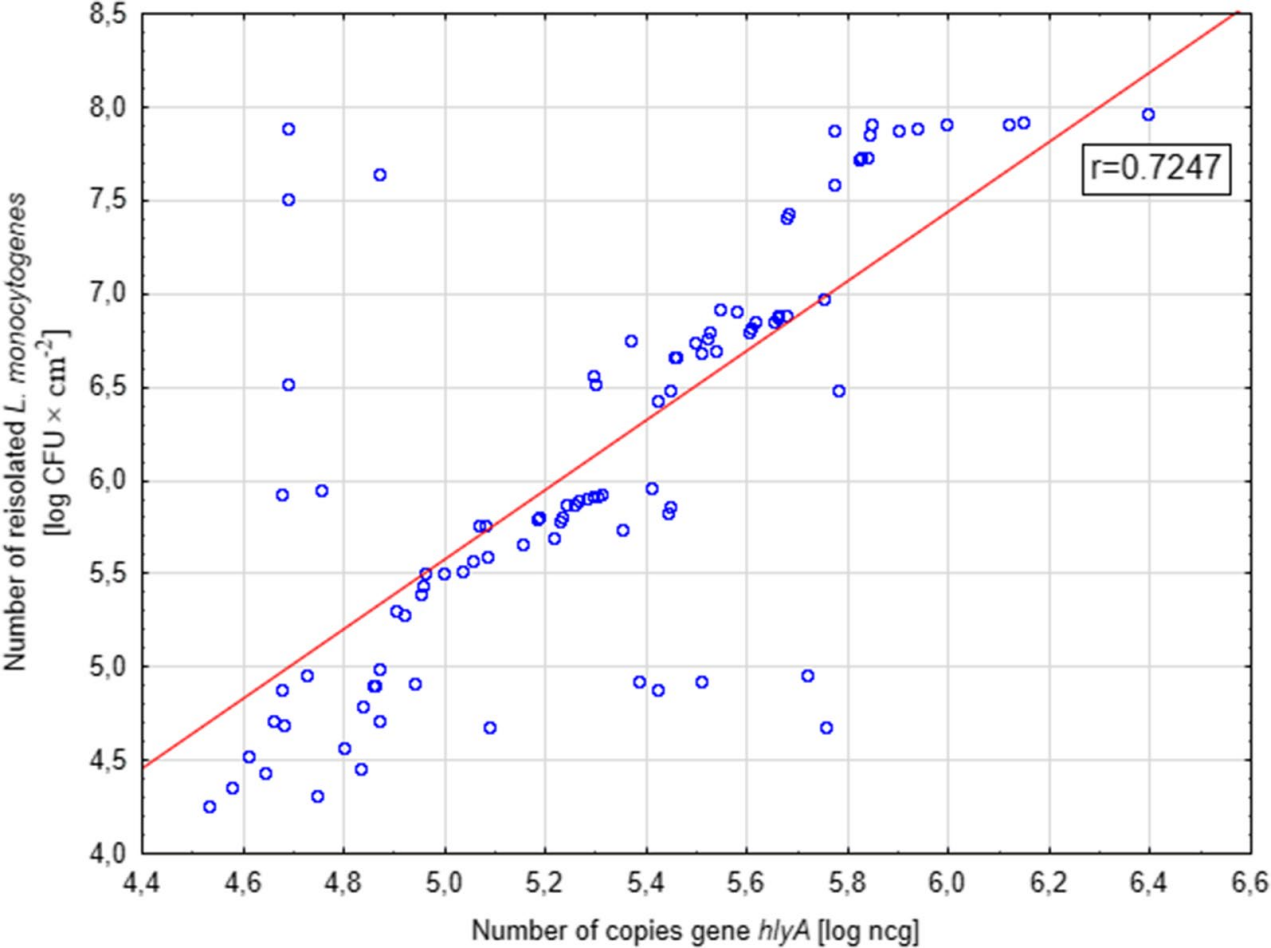
Correlation between the number of bacteria reisolated from the steel surface and the number of copies of the *hlyA* gene in the sample for the tested strains of *L. monocytogenes*

The analysis of the relationship between the number of bacteria recovered from the biofilm formed on both tested surfaces and the number of *hlyA* gene copies detected in the sample showed a high positive correlation (Figs. 3, 4).

For the biofilm formed on the AISI 304 stainless steel surface, the Pearson correlation coefficient between the classical culture-based method and the ddPCR method was 0.864 (Fig. 3). In turn, for the biofilm formed on the polypropylene surface, the correlation coefficient was 0.725 (Fig. 4). In both cases coefficients were significant (p ≤ 0.001).

## Discussion

Currently, one of the microbiological hazards is the ability of bacteria to develop biofilm, both on biotic and abiotic surfaces. In biofilm bacteria are more resistant than planktonic cells to adverse environmental conditions, including the action of antimicrobial substances such as antibiotics and disinfectants (Fagerlund et al. 2017). Due to the complex structure, biofilm eradication is a big challenge and encounters technical obstacles. Since traditional methods, based on the visualization of bacteria or determination of biofilm weight, are time-consuming there is a need for searching new methods allowing fast and precise quantification. The classical culture-based method in which colony forming units (CFU) are determined on agar medium, is commonly used in many laboratories, but has numerous disadvantages and limitations. Live bacteria detached from the biofilm layer may not be a representative part of the initial bacterial population in the biofilm. In addition, this method does not detect live bacteria not able to grow (viable but non-culturable (VBNC)) (Li et al. 2014). Digital droplet PCR seems to be a good alternative for classical culture-based methods, though this technique detects also extracellular DNA (eDNA) and DNA of dead cells, thereby possibly increasing the bacteria number (Klein et al. 2012). Therefore, it is of great importance to obtain pure and high-molecular (non-degraded) DNA (Quigley et al. 2012). In the present study, we used a column-based method (spin column-based nucleic acid purification) allowing fast and efficient DNA isolation.

The objective of the study was the comparison of classical culture-based method and a modern tool of molecular biology—ddPCR—for the assessment of the bacteria number in biofilms formed on steel and polypropylene surfaces. In the available literature these methods have not been compared so far. Klančnik et al. (2015) used ddPCR techniques for the first time to assess the number of *L. monocytogenes* in biofilm produced in microtiter plates. The specificity and sensitivity of qPCR and ddPCR assays, targeting listeriolysin O (*hlyA*) gene, have been reported previously (Klančnik et al. 2015; Traunšek et al. 2011). The *hlyA* gene is one of the most popular target gene for PCR amplification and has been shown to be specific for *L. monocytogenes* species. To date, ddPCR has been used for determination of number of foodborne pathogens (Bian et al. 2015; Porcellato et al. 2016; Suo et al. 2010), genetically modified organisms (GMO) (Koppel et al. 2015), virus HIV (Kiselinova et al. 2014), soil bacteria (Kim et al. 2014) and bacteria in surface waters (Cooley et al. 2018). It was mainly applied in studies where a small number of bacteria in the sample was expected (Porcellato et al. 2016). This method is not widely used in tests where the target molecules are abundant. Guilbaud et al. (2005) were first to use the real-time PCR method to detect and quantify *L. monocytogenes* in biofilm.

Our research showed a high positive correlation for both methods of biofilm formation assessment, on both tested surfaces. The Pearson correlation coefficient were 0.725 and 0.864 for the biofilm formed on the steel surface and on the polypropylene surface, respectively. Klančnik et al. (2015), in their research, compared various methods of DNA isolation and cell release from the biofilm layer, which may affect the effectiveness of real-time PCR. They showed that the most effective method for the isolation of cells from the biofilm, produced in the microtiter plates, was the heating of samples, 10-min sonication and centrifugation (2000×*g* for 5 min). In our research, we also applied 10-min sonication and sample centrifugation (9700×*g* for 5 min).

Bonsaglia et al. (2014) showed that the ability of *L. monocytogenes* to form biofilm is associated with the surface type on which it is formed. In our research, we did not observe statistically significant differences in the number of bacteria reisolated from steel and polypropylene surfaces. In the classical culture-based method, the number of bacteria recovered from the ASI 304 stainless steel surface ranged from 4.28 to 7.90 log CFU × cm^−2,^ whereas for the polypropylene surface ranged from 4.26 to 7.96 CFU × cm^−2^. On the other hand, the intensity of biofilm formation was strain-dependent. Environmental strains formed significantly stronger biofilm on the polypropylene surface than clinical strains. The inverse relationship was observed for biofilms produced on the steel surface—clinical strains formed the biofilm with a statistically higher intensity compared with environmental strains. Similar results were obtained with both methods used in the study. Similar relations were also observed in the research of Barbosa et al. (2013). They noted that clinical *L. monocytogenes* isolates, 70.3% (n = 83) formed biofilm of low intensity, whereas only 3.4% (n = 4) of strains were strong biofilm formers (Barbosa et al. 2013). In turn, Doijad et al. (2015) observed that 55.6% of clinical isolates (n = 10) were characterized by low biofilm formation ability and 44.4% (n = 8) were classified as moderate biofilm-forming strains (Doijad et al. 2015).

Previous studies have reported that *L. monocytogenes* forms biofilm stronger on hydrophilic surfaces such as steel than hydrophobic ones e.g. polypropylene or polystyrene (Bonsaglia et al. 2014; Chavant et al. 2002; Di Bonaventura et al. 2008). In our research, we did not observe such a correlation. Nevertheless it should be pointed out that biofilm formation is influenced by other factors like temperature (Barbosa et al. 2013), nutrients availability (Kadam et al. 2013) and biofilm formation maturity (de Oliveira et al. 2010).

In our experiment two methods assessing the strength of biofilm formation by *L. monocytogenes* were compared. We showed a high positive correlation between the classical culture-based method and ddPCR, indicating the last one as a new tool for the assessment of the bacteria number in a biofilm.

In the available literature there is no data on the application of the ddPCR technique to assess the number of bacteria isolated from the biofilm formed on various surfaces. The present study showed a high positive correlation between the results obtained by the classical culture-based method and the ddPCR technique on both tested surfaces (ASI 304 steel and polypropylene). This technique can be an alternative to traditional time-consuming methods. Nonetheless, further research is needed to optimize ddPCR as a technique for the quantification of the bacteria number in the biofilm layer.

## Data Availability

Corresponding author could provide the all experimental data on valid request.
